# Rumor Detection over Varying Time Windows

**DOI:** 10.1371/journal.pone.0168344

**Published:** 2017-01-12

**Authors:** Sejeong Kwon, Meeyoung Cha, Kyomin Jung

**Affiliations:** 1 Graduate School of Culture Technology, Korea Advanced Institute of Science and Technology, Daejeon, Republic of Korea; 2 Department of Electrical and Computer Engineering, Seoul National University, Seoul, Republic of Korea; Tianjin University, CHINA

## Abstract

This study determines the major difference between rumors and non-rumors and explores rumor classification performance levels over varying time windows—from the first three days to nearly two months. A comprehensive set of user, structural, linguistic, and temporal features was examined and their relative strength was compared from near-complete date of Twitter. Our contribution is at providing deep insight into the cumulative spreading patterns of rumors over time as well as at tracking the precise changes in predictive powers across rumor features. Statistical analysis finds that structural and temporal features distinguish rumors from non-rumors over a long-term window, yet they are not available during the initial propagation phase. In contrast, user and linguistic features are readily available and act as a good indicator during the initial propagation phase. Based on these findings, we suggest a new rumor classification algorithm that achieves competitive accuracy over both short and long time windows. These findings provide new insights for explaining rumor mechanism theories and for identifying features of early rumor detection.

## Introduction

Rumors are a powerful, pervasive, and persistent force that affects people and groups [1]. Interest in the psychology of rumors and their control has increased since World War II [2, 3], where these early studies relied on extensive yet manual data collection from books, newspapers, magazines, and interviews. Rumors have been described in numerous fashions, where the most well known definitions are ‘public communications that are infused with private hypotheses about how the world works’ [4] and ‘ways of making sense to help us cope with our anxieties and uncertainties [5]. As these definitions suggest, rumors help members of a society learn about important issues by offering a collective problem-solving opportunity to individuals who participate.

The social media era has facilitated rumor propagation even further, as any piece of information now can be promoted by online users without censorship. False rumors or unverified information can spread more rapidly and widely because individuals receiving information through this medium lack the capacity to determine the veracity of the information they receive. Some rumors have mistakenly harmed reputation of individuals or organizations and this negative role has received much attention in both research and society [6]. With the Internet and social media becoming a major information dissemination channel, rumor studies have started utilizing large-scale data [7–9] and to employ driven-intensive quantitative methods for rumor identification [10, 11]. User activity logs have been used to explain the evolutionary pattern of rumors under different conditions and to verify related social and psychological theories [12]. In addition to exploratory studies, researchers also have suggested various machine learning-based algorithms to develop rumor classifiers [13].

Most rumor classification studies are not free from two limitations. First is that the critical process of feature identification is driven by data rather than theoretical grounds, and hence there is no guarantee that findings apply for different data. To alleviate this problem, we investigated various rumor theories from social, psychological, and CMC studies and used them to derive features. While some of the rumor theories that we base on do not rely on data-driven analyses, they have been shown to be viable in explaining rumors on online communication platforms [14]. Second is that previous studies report findings based on a single snapshot of data, assuming that identified features are important throughout the rumor propagation phases. However, rumor propagation process is more complex and is known to show evolutionary patterns similar to group problem-solving activities [15]. Thus, it is more natural to assume that propagation mechanisms will change by observation periods—a characteristic that no previous data-driven study paid attention to. In the present study, we repeated statistical comparisons and classification experiments over different snapshots of 111 real rumor and non-rumor events to gain a comprehensive understanding.

Rumors in this study are defined such that a statement should (*i*) be unverified at the time of circulation and (*ii*) either remain unverified or be verified to be false after some time (i.e., at the time of this study). Hence a leak of information about later-confirmed facts (e.g., a merger or acquisition news that later gets confirmed) is not considered a rumor in this study, although its spreading pattern may look rumor-like during initial circulation. Our results provide new insight into understanding different rumor and non-rumor spreading mechanisms over time. Furthermore, we propose the best set of features and an algorithm for early rumor classification. This study makes use of longitudinal and near-complete data containing all public conversations of Twitter users over a 3.5-year period and their network structure. The data allow us to examine rumor propagation instances in the past. Rumor-related tweets were extracted from this data and examined over different time windows, form the initial 3 days to 7, 14, 28, and 56 days of circulation. Analyzing millions of rumor tweets with four sets of features from different perspectives help us quantify the relative effects of features, as follows:

The first set of features is about user themselves. Theory-driven rumor literature notes that ‘rumor spreaders are persons who want to get attention and popularity’ [16]. This hypothesis has inspired data-driven studies to assume that rumormongers have special characteristics in terms of their follower count, friend or following count, and the tweet count of users [17]. Expanding on these metrics, we consider a thorough set of the percentile, mean, variance, skewness, and kurtosis values in this research. The user-related features alone can differentiate rumors with F1 scores of 0.76 at an early stage of propagation (e.g., the first three days). Nevertheless, the classification performance decreases when the observation period becomes longer.

The second set of features is the linguistic characteristics of the rumor messages. Here, we compile an extensive list of linguistic features from previous studies and test them in rumor exploration and classification. Previous studies in the Natural Language Processing (NLP) domain have developed techniques to count the frequency of question marks, hashtags, dimensions of sentiment (e.g., positive, negative), and occurrences of arbitrary words [18, 19]. Past studies in the psycholinguistics domain on the other hand have examined the mapping between words and their semantic meanings such as social and psychological processes (e.g., tentative words such as ‘maybe’, ‘perhaps’, etc.) [20, 21]. We utilize all of these linguistic features in the context of rumors to understand why and how rumors spread. The training result demonstrates that the linguistic features are robust throughout all observation periods with F1 scores of 0.8-0.86.

The third set of features consists of structural properties of the rumor diffusion network. Networks have often been considered a key data type in general cascade studies [22–24] as well as in rumor studies [25–28]. We consider three types of propagation structures—the extended network, the friendship network, and the diffusion network—and estimate key measures such as network density and clustering coefficient. Considering these various network structures is important for obtaining features of information propagation and many complex network studies have introduced new adequate structures from theory, observation, and numerical analysis such as Fast Fourier Transformation (FFT) and the Finite Element Method (FEM) [29–31]. The diffusion network that is of the smallest in size is simple yet effective in capturing rumor information flows among users. This network helps us obtain information about nonreciprocal interactions among users. Classification result demonstrates that the network features do not perform well during initial circulation period, whereas its F1 score improves to 0.79 for a longer time duration.

Finally, we consider temporal features extracted from daily time series of the number of tweets as parameters of an epidemic model. Epidemic models have been popularly used to describe information adoption process due to their similarity in mechanisms. Among them are practical models suitable to describe non-uniform transmission and disease interactions [32, 33]. Recent models further consider new features like awareness, homophily, and diverse activity patterns with multiplexes to explain cooperative actions [34–36]. In the same manner, we identified major temporal features of each rumor (such as the natural propagation cycle) based on the Periodic External Shock (PES) epidemic model proposed in earlier work [37]. While temporal features are found to be the most predictive for longitudinal data (with an F1 score 0.88 for the 56-day window), they become unavailable when observation window is limited to the initial few days of circulation.

## Materials and Methods

We begin by discussing the methodology to extract rumor and non-rumor events for analysis. The process of feature extraction based on data observations and psychological theories about rumor spreading are introduced.

### Rumors on Twitter

The massive data logged in social media provide an opportunity to study rumors quantitatively. One of the largest and near-complete repositories of Twitter is from [38], where the authors crawled the network and took a snapshot of the 54 million public profiles and 1.9 billion follow links among them in August 2009. The repository also contains the 3200 most recent tweets of every individual at the time (which nearly covers the complete tweet history of the individuals, since the launch of Twitter in 2006) accounting a total of 1.7 billion tweets. This longitudinal data were used to extract popular rumors between 2006 and 2009, which reveal various features such as the user-level, structural, temporal, and linguistic characteristics at the time of circulation.

To select rumor cases, we searched for two famous rumor archives, snopes.com and urbanlegends.about.com and identified popular rumors at the time of the dataset. For non-rumor cases, we searched for notable events from news media outlets like times.com, nytimes.com, and cnn.com. We identified a total of 130 events (72 rumors and 58 non-rumors) from the time period covered by the Twitter dataset and extracted tweets corresponding to these events based on two criteria: (1) a tweet should contain explicit keywords relevant to the event and (2) a tweet should have been posted during the time of circulation (i.e., within the first two months).

To test whether tweets identified in the above manner are indeed relevant to the event of interest, we hired four human labelers to evaluate the dataset. The labelers were asked to judge whether each event was rumor or non-rumor by examining four randomly selected tweets and URLs embedded in tweets for each event. Then, we selected the events that were evaluated by four participants and had the majority agreement for this study. The final set of rumor and non-rumor events had agreement among three or more labelers. The intra-class correlation coefficient (ICC), which measures the level of agreement, was 0.993 and the p-value was close to zero. As a result, 111 events were retained (60 rumors and 51 non-rumors). S1 and S2 Tables list the selected rumors and non-rumors, respectively.

The key contribution of this paper is at examining rumor characteristics over different observation time windows, from the initial 3, 7, 14, and 28 to 56 days of circulation. For each of these time windows, we repeatedly estimated features from user, linguistic, network, and temporal characteristics. Once all feature values were obtained, we traced the changes in the statistical differences and predictive powers of these features in distinguishing rumors from non-rumors.

### User Feature

The question of who spreads rumors has been investigated extensively in previous qualitative studies through interviews and user surveys. A particular focus has been on user influence [39]. Because rumors have the power to arouse curiosity, individuals who seek attention can exploit rumor rhetoric [16]. In contrast, the low credibility of rumors can hurt reputation individuals who participate in the discourse and hence highly influential individuals may try to avoid participating in rumor conversations [37]. These findings hence suggest that rumor spreaders typically involve individuals with small or moderate influence on social networks.

**Fig 1:**
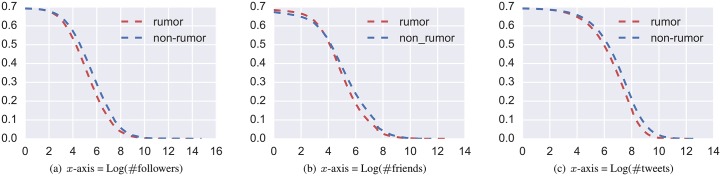
Log-log scale of the CCDF. Complementary Cumulative Distribution Function (CCDF) of aggregated user characteristics for rumor and non-rumor events in the 56-day observation.

We consider the number of followers, friends, and tweets as a proxy of user influence. Fig 1 illustrates the distributions of these three quantities aggregated over rumor and non-rumor events, respectively. The figure shows that, across the majority of quantile values, user features for non-rumor events are higher than those of rumor events, suggesting that user features can be used for determining rumors from non-rumors. In order to represent this difference, we approximated the rumor distributions at the 25th, 50th, 75th, and 100th percentiles, standard deviation, skewness, and kurtosis values and utilize these for rumor classification. S3 Table lists the 27 variables for describing the user characteristics for each event.

### Linguistic Feature

Previous studies that develop linguistic features have primarily focused on building classifiers of high accuracy but have not provided supportive theories regarding why the proposed features work [7, 17, 40]. In contrast, we start from theory-based rumor research to define relevant linguistic features that are missed in data-driven studies. Psychological theories can explain why and how people react to a given rumor [39, 41]. For example, upon receiving a rumor, a person will first raise a doubt, judge its meaning with her knowledge, then check with existing sources to verify its veracity [42]. This process of doubt ends when a person gathers sufficient evidence, at which point the user either accepts the rumor by propagating it further or rejects it by either ignoring it or expressing negating comments. We define features of doubt, negation, and guessing to describe this process.

Reputation is also known to give people a strong incentive to conform to social norms in this process [43]. Low credibility of rumors and doubts incurred by rumor audience will result in a different writing style of rumor conversations compared to conversations on non-rumors. For example, rumors are more likely to contain words related to cognition (e.g., skepticism, guessing) than non-rumors. Furthermore, because rumors are known to propagate among social peers via word-of-mouth (than involving authorities as in non-rumors) [5], they are more likely to referring to language on social relationships (e.g., family, mate, friend), and actions like hearing. We also define features related to social processes and cognition.

Below are example rumors on a fake celebrity death news that use such language:

(Tweet 1) Emma Watson died in a car accident last night, but I’m not gonna believe it unless BBC reports it. It’s all REALLY fishy.

(Tweet 2) Nope, it’s supposedly a hoax because they were claiming that Harrison Ford had died as well. Not true.

As seen in these examples, words related to skepticism (‘hoax’), negation (‘not true’), and doubt (‘not gonna believe’) appear. Rumor tweets are sometimes written in capital letters as shown above, which is a way of shouting or adding emphasis online. Below is another type of rumor regarding health issues, which also uses capital letters and a sign of doubt. The rumor below also confirms another theory about rumors, which is unclear information sources (‘heard on’).

(Tweet 3) Just heard on the radio…. There is NO link between breast cancer and deodorants.

We utilize a sentiment analysis tool, Linguistic Inquiry and Word Count (LIWC), which was built to identify a group of words that have basic emotional and cognitive dimensions that are often studied in social, health, and personality psychology. Its dictionary has approximately 4,500 words and word stems each of which is assigned to several predefined categories. In order to compute sentiment scores for given text, each word in the text is compared with the LIWC dictionary. Considering the example in Tweet 1, the program would find the word ‘not’ and verify whether this word is in the negation category. Then, the score of the negation category will increase. If the score of the negation category is 2.34, this means that 2.34% of all words in the given text matched with the negation category. There are five major categories and a number of subcategories about psychological processes (e.g., social, affect, cognitive, perceptual, tentative, swearing) and writing patterns (negation, home, tense), which satisfy the requirements to examine the hypotheses in this study.

Prior to analysis, we sanitized the tweet data by removing ‘@username’, hashtags, short URLs, as well as any emoticons and special characters that the LIWC tool cannot parse. The tool’s guideline suggests to ensure at least 50 words for analysis. Given that individual tweet is short in length, we grouped tweets belonging to the same event into a single input file and measured the LIWC sentiment scores collectively for each event.

### Network Feature

Network representation enables the entire propagation pattern of information and interactions among users to be visualized [12, 44]. We defined three kinds of structures—the extended network, the friendship network, and the diffusion network.

**Fig 2:**
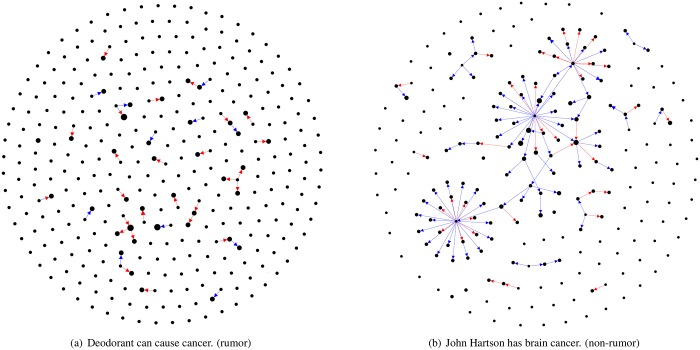
Diffusion network examples. The network visualization in (a) shows that rumors involve a larger fraction of singletons and smaller communities, resulting in a sporadic diffusion pattern. In contrast, the diffusion network of a non-rumor event in (b) is highly connected, forming a giant connected component and a smaller fraction of singletons. Edge colors represent the relative influence of the spreader and recipient, such that red (blue) means information propagated from a lower-degree (higher-degree) spreader to a higher-degree (lower-degree) recipient.

The extended network is defined as an induced directed subgraph of users, who have either posted any tweet related to the event or who is following or is followed by other participating users, from the original follower-followee relationship on Twitter. The friendship network is a smaller induced subgraph of the extended network that only contains users who have tweeted content related to the event and the links among such users. The diffusion network is a subgraph of the friendship network, which depicts the direction of information flow among users. Information flows are defined such that a event is diffused from user A to user B, if and only if (1) B follows A on Twitter and (2) B posted about the given event by mentioning the appropriate keywords only after A did so. When a user has multiple possible sources of flows, we select the user who most recently posted about the event as the source. Then, we retain the links of the selected source and the target in a diffusion network, which hence captures information flows. Fig 2 presents the diffusion networks of a rumor and non-rumor events, respectively. These networks were extracted from the 56-day observation period.

Network measures were computed from the three kinds of networks explained above. In addition to the traditional measures such as density and clustering coefficient, we also considered measures that are specific to rumor propagation. For instance, rumors are known to spread without strong evidence and have low response rate, in particular when many rumor receivers decide not to participate in the conversation [43]. Such process can be captured by the count or the fraction of singletons in the friend network. Consequently, each network and its largest connected component have an identical set of features, such as the average clustering coefficient, density, the number of nodes, the number of edges, and the fraction of users with nonreciprocal interactions.

Not only standard metrics but also rumor-specific measures (inspired by theoretical studies on rumors) were defined for this study. For these measures we rely on the diffusion network, whose direct links represent inferred information flows. One example is the direction of information flow. In rumor propagation, influential users are known to restrain themselves from participating; hence initial rumor conversations will begin from individuals with relatively low or medium influence and then ultimately reach a wider set of the networks [37]. This process was captured by examining, for each information flow instance, whether the direction of flow started from a lower-degree user to a higher-degree user. S4 Table presents the comprehensive list of 43 network features that were analyzed.

### Temporal Feature

**Fig 3:**
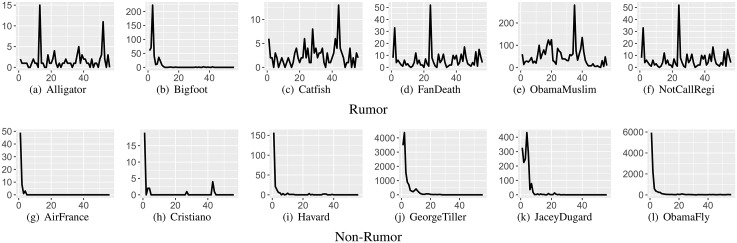
Samples of extracted time series. The time series are extracted from 56-day observation period(*x*-axis = days; *y*-axis = number of tweets). Rumors typically have longer life spans and more fluctuations.

A rumor characteristic that has received recent attention is the temporal aspect. Fig 3 depicts several time series of rumor and non-rumor events, which shows multiple and periodic spikes for rumor events. In contrast, most non-rumor events appear with a single prominent spike. While non-rumor events typically show a large peak during the initial phase of circulation that decays quickly over time, rumor events are known to show repeated peaks over time that lead to a cyclic trend. Such cyclic behavior can be captured mathematically by the Periodic External Shock (PES) model [37], which is our previous work that is an extension of the time-series fitting model proposed in [45]. Here, we describe how we derived the PES model from the base model. The base model, called SpikeM, is from the Susceptible-Infected (SI) model in epidemiology and captures both the periodic behaviors and power-law decays that are popularly observed in real-world data. The SpikeM model is formally described as below.

*SpikeM with parameters* *θ* = {*N*, *β*, *n*_*b*_, *S*_*b*_, *ϵ*, *p*_*p*_, *p*_*a*_, *p*_*s*_}:

$$\begin{array}{ccc} & & \;U(n+1)=U(n)-\Delta B(n+1),\\ & & \Delta B\left(n+1\right)=p\left(n+1\right)\cdot [\frac{\beta }{N}\cdot U\left(n\right)\cdot \\ & & \;\sum_{t=n_{b}}^{n}\left(\Delta B\left(t\right)+S\left(t\right)\right)\cdot {\left(n+1-t\right)}^{-1.5}+\epsilon ] \end{array}$$ (1)

$$\begin{array}{ccc} p(n) & = & 1-\frac{1}{2}p_{a}[1+\sin(\frac{2\pi }{p_{p}}\left(n+p_{s}\right))],\\ S(t) & = & S_{b}\;\text{when}\;t=n_{b};\;\text{otherwise}\;0.\\ & & \;U(0)=N,\Delta B(0)=0. \end{array}$$

In the model, *U*(*n*) is the number of uninfected (or uninformed) nodes in the network at time step *n*; Δ*B*(*n*) is the newly infected (or informed) nodes at time *n*; *N* is the total number of nodes involved in the diffusion process; *n*_*b*_ is the time when the first external shock of the event occurs, and *S*_*b*_ is the scale of the first external shock, i.e., the number of nodes that are infected at the beginning of the event at time *n*_*b*_. Thus, the term $\sum_{t=n_{b}}^{n}\left(\Delta B\left(t\right)+S\left(t\right)\right)$ represents the total number of infected nodes at time *n*. Then $\Delta B\left(n+1\right)=\frac{\beta }{N}\cdot U\left(n\right)\cdot \sum_{t=n_{b}}^{n}\left(\Delta B\left(t\right)+S\left(t\right)\right)$ is the standard SI model describing that at time *n* + 1 each uninfected node *u* randomly selects another node *v* from all nodes and if *v* is already infected; *u* becomes infected at time *n* + 1 with the probability *β*, which is a parameter for infection strength.

The SpikeM model extends the SI model through introducing (a) a power-law decay term (*n* + 1 − *t*)^−1.5^ in Eq (1) so that the strength of the infection of previously infected nodes becomes weaker in a power-law decay pattern, and (b) a periodic interaction function *p*(*n*) that reflects people’s periodic interaction patterns (e.g. people may have more time to interact on Twitter in the evening than during the day when they are at work or school). Parameters *p*_*p*_, *p*_*a*_, and *p*_*s*_ correspond to the period, amplitude, and phase shift of the periodic interaction function, respectively. Finally parameter *ϵ* represents a noise term in the model.

While the SpikeM model can adequately capture any periodic or seasonal patterns in real-world data, the model is not directly applicable for rumor analysis. This is because the multiple peaks seen in rumors are not due to people’s natural tendency to repeat behaviors daily or weekly (shown as the periodic interaction function *p*(*n*)), but is more irregular and may be driven by a number of external factors. For example, peaks may appear when a rumor reaches a new set of audience who were not exposed to the same information previously or when a group of rumor mongers decide to spread information further.

We hence extended the SpikeM model and proposed the Periodic External Shocks (PES) model, which newly assumes there may be *multiple* external shocks and each shock will show a power-law decay over time in [37]. For simplicity, external shocks are assumed to have a short periodic cycle. Then the PES model is described as below:

*PES model with parameters* *θ*′ = {*N*, *β*, *n*_*b*_, *S*_*b*_, *ϵ*, *p*_*p*_, *p*_*a*_, *p*_*s*_, *q*_*p*_, *q*_*a*_, *q*_*s*_}:

$$\begin{array}{ccc} & & \Delta B\left(n+1\right)=p\left(n+1\right)\cdot [\frac{\beta }{N}\cdot U\left(n\right)\cdot \\ & & \;\sum_{t=n_{b}}^{n}\left(\Delta B\left(t\right)+\bar{S}\left(t\right)\right)\cdot {\left(n+1-t\right)}^{-1.5}+\epsilon ], \end{array}$$ (2)

$$\begin{array}{ccc} \bar{S}\left(t\right) & = & S(t)+q(t),\\ q(t) & = & q_{a}[1+(\sin(\frac{2\pi }{q_{p}}\left(t+q_{s}\right)))],\\ & & \text{All other terms are the same as in SpikeM}. \end{array}$$

The PES model has a periodic external shock function *q*(*t*), which has three parameters *q*_*p*_, *q*_*a*_ and *q*_*s*_ to represent the period, amplitude, and shift of the periodic external shock function, respectively. When *q*_*a*_ = 0, the PES model is reduced to SpikeM and hence can be considered a generalization of the SpikeM model. Initial shock function is termed *S*(*t*). We consider the parameters of the PES model as temporal features of rumors. For parameter learning, the Levenberg-Marquard method [46] was used to minimize the sum of the squared errors: *D*(*X*, *θ*) = ∑_*n*_(*X*(*n*) − Δ*B*(*n*))^2^ with a given time series *X*(*n*). S5 Table displays the final set of temporal features.

## Results

### Changes in significance over time

In order to identify the significant differences between rumors and non-rumors for the first 3, 7, 14, 28 and 56 days from the initiation, Mann-Whitney U test is used. Tables 1–4 list the user, network, linguistic and temporal features with constant tendencies over these observation periods.

**Table 1:** User features with invariant tendency during the observation periods.

Symbol	Description	Class				
		3	7	14	28	56
*m*_*fr*_	Minimum of number of friends	R	R	R	R	R
*q*75_*fr*_	75th percentile of number of friends	N	N	N	N	
*M*_*fr*_	Maximum of number of friends	N		N	N	N
*σ*_*fr*_	Standard deviation of number of friends	N	N	N	N	N
*q*25_*fo*_	25th percentile of number of followers	N	N	N	N	N
*med*_*fo*_	median of number of followers	N	N	N	N	N
*q*75_*fo*_	75th percentile of number of followers	N	N	N	N	N
*M*_*fo*_	Maximum of number of followers	N	N	N	N	N
*μ*_*fo*_	Average of number of followers	N	N	N	N	N
*σ*_*fo*_	Standard deviation of number of followers	N	N	N	N	N
*κ*_*fo*_	Kurtosis deviation of number of followers	N	N	N	N	N
*S*_*fo*_	Skewness of number of followers	N	N	N	N	N
*q*25_*t*_	25th percentile of number of tweets	N	N	N	N	N
*med*_*t*_	median of number of tweets	N	N	N	N	N
*q*75_*t*_	75th percentile of number of tweets	N	N	N	N	N
*M*_*t*_	Maximum of number of tweets	N	N	N	N	N
*μ*_*t*_	Average of number of tweets	N	N	N	N	N
*σ*_*t*_	Standard deviation of number of tweets	N	N	N	N	N

In the Class column, fields denoted with R (or N) indicate that the target feature over the specific observation period had higher value for rumors (or non-rumors) at a significant level of *p* < 0.05 based on the Mann-Whitney U test. A Blank field indicates that there was no significance in correlation. Results are shown for observation periods of 3, 7, 14, 28, and 56 days.

**Table 2:** Linguistic features with invariant tendencies during the observation periods.

Symbol	Description	Class				
		3	7	14	28	56
family	family (daughter, father, husband, aunt)	N	N	N	N	N
i	1st person singular (I, me, mine)	R	R	R	R	R
you	2nd person (you, your, thou)	R	R	R	R	R
conj	conjunctions (and, but, whereas, although)	R	R	R	R	R
present	present tense (is, does, hear)	R	R	R	R	R
auxverb	auxiliary verbs (am, will, have)	R	R	R	R	R
discrep	discrepancy (would, should, could)	R	R	R	R	R
adverb	adverb (very, really, quickly)	R	R	R	R	R
excl	exclusive (but, without, exclude)	R	R	R	R	R
cogmech	cognitive mechanism (cause, know, ought)	R	R	R	R	R
negate	negations (not, no, never)	R	R	R	R	R
tentat	tentative (maybe, perhaps, guess)	R	R	R	R	R
assent	assent (agree, OK, yes)		R	R	R	R
certain	certain (always, never)		R	R	R	R
social	social processes (mate, talk, they, child)			R	R	R
swear	swear words (damn, piss, f*ck)			R	R	R
hear	hear (listen, hearing)			R	R	R

The ‘Description’ column lists example words for each symbol.

**Table 3:** Network features with invariant tendencies during the observation periods.

Symbol	Description	Class				
		3	7	14	28	56
*V*_*e*_	Number of nodes of extended network	N	N	N	N	N
*E*_*e*_	Number of edges of extended network	N	N	N	N	N
*NI*_*e*_	Number of nodes without incoming edge in extended network	N	N	N	N	N
*NO*_*e*_	Number of nodes without outgoing edge in extended network	N	N	N	N	N
*D*_*el*_	Density of LCC of extended network	R	R	R	R	R
*E*_*el*_	Number of edges of LCC of extended network	N	N	N	N	N
*V*_*f*_	Number of nodes of friendship network	N	N	N	N	N
*E*_*f*_	Number of edges of friendship network	N	N	N	N	N
*NO*_*f*_	Number of nodes without outgoing edge in friendship network	N	N	N	N	N
*D*_*fl*_	Density of LCC of friendship network	R	R	R	R	R
*E*_*fl*_	Number of edges of LCC of friendship network	N	N	N	N	N
*pNI*_*f*_	Proportion of nodes without incoming edges of friendship network	R	R	R	R	R
*pNO*_*f*_	Proportion of nodes without outgoing edges of friendship network	R	R	R	R	R
*pI*_*f*_	Proportion of isolated nodes of friendship network	R	R	R	R	R
*D*_*dl*_	Density of LCC of diffusion network	R	R	R	R	R
*V*_*dl*_	Number of nodes of LCC of diffusion network	N	N	N	N	N
*E*_*dl*_	Number of edges of LCC of diffusion network	N	N	N	N	N
*pNI*_*d*_	Proportion of nodes without incoming edges of diffusion network	R	R	R	R	R
*pNO*_*d*_	Proportion of nodes without outgoing edges of diffusion network	R	R	R	R	R
*pI*_*d*_	Proportion of isolated nodes of diffusion network	R	R	R	R	R

**Table 4:** Temporal features with invariant tendencies during the observation periods.

Symbol	Description	Class				
		3	7	14	28	56
*q*_*p*_	Periodicity of external shock				N	N
*q*_*s*_	External shock periodicity offset			N	N	N
*p*_*s*_	Interaction periodicity offset			N	N	N

Table 1 focuses on the significant features among the user characteristics. Considering the number of followers and tweets, non-rumors had higher values for all quantiles except the minimum. This can be interpreted that users participating in non-rumor spreading are more active tweeters and have larger audiences. Kurtosis and skewness of the number of followers are also higher for non-rumors, which indicates that non-rumors are more likely have users with extraordinarily larger audiences. These observations lead to a conclusion that users who get more attention on Twitter (i.e., have more followers) are less likely to participate in rumor spreading. This observation provides a meaningful insight that the high audience size of users might hinder a user from participating in rumor propagation in addition to the existing findings about effects of audience size from other information propagation studies [38].

The minimum number of friends (*m*_*fr*_) is the only a feature, whose values turn out higher for rumors than non-rumors. For validation, we examined which individuals had the smallest number of friends for each event. We found non-rumor events to involve news media accounts like ‘BBCNewsOnceADay’, which have large followers and disproportionately small followings (i.e., friends). At the time of circulation, the existence of such media with a small number of friends and a large number of followers creates the particular propagation patterns that rumors have higher minimum number of friends but lower values for the others.

Table 2 supports the assumptions about the different cognitive and writing styles for rumormongers described in the social and psychological theories. According to Table 2, it can be seen that rumors have more words related to a certain cognitive style such as skepticism. Unlike other types of information, users who are more likely spread not only the story but also their opinion and thinking about a rumor. New patterns began to appear after 7 days. Users began to mention social relationships (e.g, mate, friend) as they referred to information sources and hearing actions (e.g., heard) in rumor conversations. We also see features with unexpected patterns. Words in assent (e.g., agree, okay), swear (e.g., damn, piss), and certain (e.g., always, never) category become significant after 14 days.

**Fig 4:**
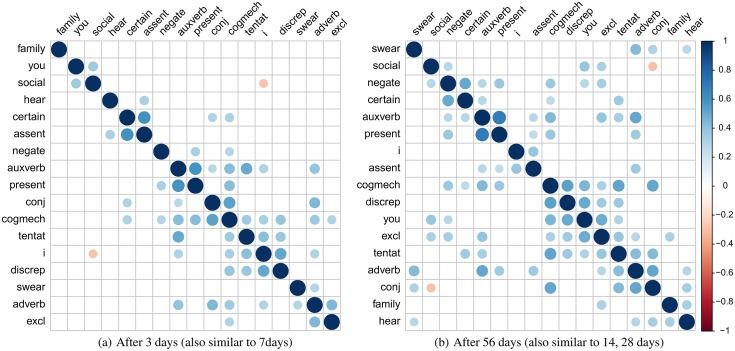
Correlogram among the linguistic features. These plots show correlations among linguistic features with significance for rumor events. Colors of circles represent correlation coefficients, where the dark blue (red) color indicates coefficients of 1 (-1). Size of circles represent the absolute values of correlation coefficients. Blank or no circle indicates non-significant cases where p-values ≥ 0.05. From (b), strong negation is expressed by a positive correlation between scores of ‘negation’ and ‘certain’. Interestingly, the ‘assent’ category increases for rumors, which indicates that users who confirmed the rumor also appeared as time passed.

Fig 4 explains why these changes occurred. As seen in Fig 4, the reactions to rumors became more confident. The first reaction is strong denial expressed by a high correlation between negation and certain. Next, simple agreement is described by the sole existence of assent category. This result can be one of the additional supportive result for Bordia’s work [15] that people express fears and anxieties or hopes and wishes in the context of the rumors.

From Table 3, counting the measures of the introduced networks such as number of nodes, edges, and certain types of user exhibited significantly higher values for non-rumors. This can be explained from the previous observation that rumor spreaders apparently have fewer followers. For non-counting measures, density of the largest connected component (LCC), proportion of users without followers (i.e., singletons), friends, or both are higher for rumors. From these findings, three propositions can be inferred: i) a rumor is more likely to be obtained from an external source, ii) a rumor is more likely to be ignored on social media even when a friend mentions it, and iii) the community structure of rumor spreaders is smaller but denser in general than those of non-rumors.

Table 4 shows that there is no temporal feature with a significant difference at the initial time. Nonetheless, for 14 days and longer duration, temporal feature about periodicity becomes significant and exhibit very high prediction performance (especially for the 56-day period), which we will discuss in more detail. This phenomenon is expected since the proposed model contains two trigonometric functions to explain the information propagation and as a result short observation periods like 3 or 7 days are not sufficiently long enough to fit the temporal model.

### Predictive Features and Classification

Machine learning was used to test the significance in differences between the rumor and non-rumor events based on the theory-driven features discussed in the previous section. A three-step variable selection process proposed in [47] was applied to identify predictive features and build a classifier. This process works as follows: first, eliminating the irrelevant variables with a small permutation importance index, second, selecting all variables related to the response for interpretation purpose (i.e., interpretation set), and third, refining the selection through eliminating redundancy in the set of variables selected in the second step, for prediction purpose (i.e., prediction set). We repeated the process 10 times and used features that appeared more than eight times in order to prevent inadvertent selection of irrelevant features due to the permutation. Hence, features that are robust discriminating factors can be selected for rumor classification.

**Fig 5:**
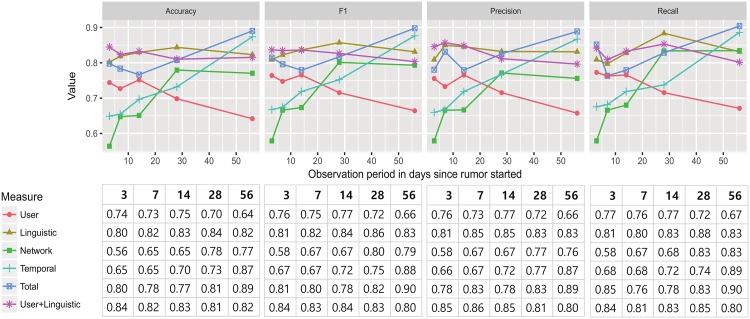
Comparison of the strength of the features in determining rumors. Total and User+Linguistic are the newly proposed rumor classification algorithms in this study.

The feature selection process was repeated for the user, linguistic, network, and temporal domains in order to compare the prediction power of each feature. With the features in the prediction sets, we applied a random forest with three-fold cross-validation. Given that the size of events (111 rumor and non-rumors) can be insufficient to conclude the prediction performance, the comparison process was repeated ten times. Fig 5 illustrates the average classification performance of each set of features according to time. Combining Fig 5 and Table 5, following observations are noted. The final outcome is summarized in Table 5, which lists the selected features for the interpretation and prediction task. We make the following observations.

**Table 5:** Selected features.

Symbol	Description	Selection				
		3	7	14	28	56
*σ*_*fr*_	Standard deviation of number of friends	**				
*σ*_*fo*_	Standard deviation of number of followers	**				
*μ*_*fo*_	Average of number of followers	**				
i	1st person singular (i, me, mine)				*	*
conj	conjunctions (and, but, whereas, although)			**	**	**
auxverb	auxiliary verbs (am, will, have)		**		**	**
adverb	adverb (very, really, quickly)		**	*	**	**
excl	exclusive (but, without, exclude)	**	*	**	**	**
cogmech	cognitive mechanism (cause, know, ought)			*	**	**
affect	affective processes (happy, cried, abandon)		**		*	
negate	negations (not, no, never)	**	**	**	**	**
tentat	tentative (maybe, perhaps, guess)	**	*	**	**	**
certain	certain (always, never)				**	**
hear	hear (listen, hearing)				*	**
*E*_*el*_	Number of edges of LCC of extended network			**	**	*
*AC*_*fl*_	Average clustering coefficients of LCC of friendship network				*	*
*pI*_*d*_	Proportion of isolated nodes of diffusion network					**
*pLTH*	Fraction of LTH among information diffusion				**	**
*pHTL*	Fraction of HTL among information diffusion				**	**
*S*_*c*_	Strength of external shock at birth				*	**
*q*_*p*_	Periodicity of external shock					**
*q*_*s*_	External shock periodicity offset					**
*p*_*s*_	Interaction periodicity offset				**	*

This table lists the features that are selected as prominent differentiating ones among all features. In the Selection column, * and ** signs indicate that the target feature was selected for the interpretation set and prediction set. If a feature is selected for the prediction set, it is also in the interpretation set by definition. A blank field indicates that the corresponding feature was not selected.

First, user features are only predictive during the initial rumor circulation. The observation that only the standard deviation matters for the classification could indicate that the existence of users with extraordinarily high numbers of followers and friends, i.e. outliers, is the most important factor in identifying rumors. On the other hand, distributions for rumors and non-rumors related to the user characteristic become similar over time as more users participate in the information propagation process. For example, if influential users mention rumors about the death of a famous person or an urgent health issue, they later deny them with confirmatory evidence. This demonstrates that certain rumor characteristics become prominent at different course of the propagation.

Second, linguistic features are powerful and stable predictors of rumors. During initial circulation, rumors can be identified by the use of words related to negation and skepticism. Over time, linguistic characteristics persist and additional patterns such as strong negation or hearing appear. Linguistic features consistently differentiate rumors with high performance (F1 score: 0.8-0.86). Furthermore, utilizing the linguistic features alone yields the higher predictive power even than using the full list of features, except for the 56-day observation window.

Third, the network features require a longer time period and further information on spreading to become predictive. The performance with short observation periods is comparable to random guessing. Only the features representing the specific direction of information flow, unique users whose source is outside the network and the rumor content being neglected are important for classification. From the results, tracing each information flow is significantly more important than knowing the entire relationship among users. Collecting the entire network structure has always been an issue in studies because it requires significant resources compared with other types of information. This can be an indicator that these expensive features are not required in the early stages of rumor classification.

Considering the temporal features, the proposed PES model excelled in capturing the prominent differences between rumors and non-rumors for the 56-day observation window. Yet, the temporal features demonstrated poor differentiating power for shorter time windows. Because the PES model to fit temporal features utilize two trigonometric functions to explain user interaction and information propagation processes, all but the 56-day observation were not sufficiently long enough to define such features.

We propose two new algorithms for rumor classification. The first algorithm, which takes every feature into account, showed good performance with F1 score in the range from 0.77 to 0.90 after 28 days from the first date of rumor spreading. This algorithm however did not perform well for shorter observation periods. For shorter time duration, regarding network and temporal features rather decreased the performance of the classifier since the performances of these features are just slightly higher than random selection. Despite the insufficient performance at early rumor detection, this algorithm showed undoubtedly good performance when the network and temporal features become well available (i.e., after 14 days).

The second algorithm is geared toward early rumor classification and considers user and linguistic features. For short observation periods, this algorithm showed better performances compared to using a full set of features. For longer time windows, its performance slightly decreased to F1 score of 0.80. This algorithm nonetheless has two strengths. First, its classification performance is less sensitive to observation periods compared to the full model. Second, it does not require any temporal or network features that rely on near-complete data obtainable only via extensive gathering or explicit APIs. Given that most social networks do not grant researchers to obtain full data, the ability to design an algorithm that detects rumors with locally obtainable information is essential for practical use.

## Discussion

The present study examined an extensive set of features related to the user, linguistic, network and temporal characteristics of rumors and compared their relative strength in classifying rumors over time. This comprehensive study was conducted on real rumor cases extracted from near-complete data of Twitter. The defined features and experiments, which draws upon existing social and psychological theories as well as unique observations from data, provides a deep understanding of rumor mechanisms as follows. First, this study identifies an early marker of rumors that is visible from the first few days of circulation; rumor participants have fewer followers and express doubt and skepticism. Second, the network and temporal features became remarkable for over two months; rumors have low adoption rates and appear repeatedly over time, which results in smaller yet more densely-knit communities compared to non-rumor networks. Third, we proposed a practical algorithm that does not require full snapshot of the network nor complete historical records. While the most predictive features of rumors changed depending on the observation window, the combination of user and linguistic features functioned well consistently over short and long time windows.

Unlike the current study, many previous studies have used a single observation window that is often arbitrarily set [7]. Results obtained from one stationary viewpoint may be insufficient to represent the general rumor spreading patterns. Instead, our work was inspired by a few studies that have argued rumor spreading mechanisms change over time [13, 14], which is a theme that we explored deeply in this paper. Nonetheless, this study shares limitations of previous rumor studies in that statistically meaningful predictive features are observational and they cannot be generalized to all rumor cases. Toward handling this limitation, we have carefully made the following efforts. First, we considered the pre-existing rumor theories from the social and psychological fields as important evidence in deriving the features and explaining the observations of the experiment. Hence, we have supportive reasons regarding why our findings are not inadvertent results. Second, we repeated the experiments for various observation periods and used only observations with invariant patterns. These efforts guide us to arriving at a more robust and reliable understanding of rumor spreading mechanisms in online social media.

The classification experiments remarkably showed that certain rumor characteristics become prominent at different course of the rumor circulation and hence appear with strong predictive power. The results provide valuable observations for identifying practical features for developing a rumor classifier. The introduced sets of features prove their validity as predictors with the minimum F1 score of 0.8. Although the time window over which each feature set showed the best performance differed, which makes it difficult to determine when a set of features becomes promising or unpromising due to the different speed of information spreading. Nonetheless, each set of features exhibited almost uniform increasing and decreasing patterns and it could be determined which feature sets are prominent for early stage rumor detection. At the rumor initiation, the linguistic and user characteristics are important for classifying rumors from streams of content.

Recent studies have begun to focus on rumor detection within only short time periods like 24 hours and they have achieved good classification results. One study highlighted the usefulness of content-based features (i.e, linguistic features) through demonstrating great performance (F1 score: 0.89) with a combination of TF-IDF (term frequency–inverse document frequency) of top-k vocabularies from data and deep learning [48]. Due to the nature of machine learning, however, there is always an issue of bias error. The linguistic features proposed in this study were drawn from a set of words that are the most common and widely used to express a person’s ideas or thinking. Thus, we expect that these linguistic features are less likely to be affected by field-specific lexis that causes bias in typical NLP methods. Combining the user and linguistic features, we plan to develop a rumor classifier that is appropriate for only small and initial fraction of content and is less sensitive to the type of information.

## Supporting Information

S1 Dataset Tweets and user characteristics for rumor and non-rumor events. See the respective attached file.
(7Z) Click here for additional data file.

S1 Table Description of non-rumor events. (PDF)

S2 Table Description of rumor events. (PDF)

S3 Table User features. Kurtosis is a measure of how much a given data is heavy or light-tailed compared to a normal distribution. Data with high kurtosis likely to have heavy tails or outliers. Skewness is a measure of symmetry. In definition column, a term ‘SD’ mean for standard deviation.
(PDF)

S4 Table Network features. In definition column, *E*, *F*, and *D* mean for extended network, friendship network and diffusion network, respectively.
(PDF)

S5 Table Temporal features. Symbols are for parameters of suggested PES model.
(PDF)
