# Supplementary material for: Rumor Detection over Varying Time Windows
Source: PLoS One. 2017 Jan 12;12(1):e0168344. doi: 10.1371/journal.pone.0168344 (PMC5230768; doi:10.1371/journal.pone.0168344)
Supplement: S1 Table — (PDF) [file pone.0168344.s002.pdf]

S1 Table. Description of non-rumor events used for analysis

Name	Description	#Tweets
N_Airfrance	Air France jet mission with 228 people over Atlantic after running into thunderstorms.	61
N_Airliner	Pilot hailed for 'Hudson miracle': The pilot of an airliner that ditched in New York's Hudson River.	116
N_Amanda	Amanda Knox to Take Stand in Murder Trial.	2593
N_AnnieLe	Body of missing Yale student Annie Le found on campus in medical school lab.	145
N_BarnesNobleObama	Barnes and Noble Store Window Features Obama Alongside Monkey Book.	39
N_BeefProtest	There are protests in Korea as American beef is about to come back on the menu.	54
N_breastMilkIceCream	Ben and jerry's breast milk ice is on sale.	372
N_ByrdBillings	Couple who adopted 12 children shot to death: Byrd and Melanie Melanie Billings were found dead.	372
N_CharlieWilsonWar	Reviews and status updates for comedy drama film, 'Charlie Wilson's War'.	3958
N_ChristianTheLion	Reviews and viewers' emotion for a video meme, 'Christian the Lion'.	3650
N_ClarkRockefeller	Rockefeller poser gets up to 5 years for kidnapping.	1079
N_Cristiano	Cristiano Roaldo lucky escape from crash in his Ferrari.	162
N_Dell	Dell enters into smartphone market.	767
N_District	Reviews and status update about a movie, District 9.	9786
N_eee1101ha	Information and reviews about a netbook, eee1101ha.	930
N_ElephantPaint	A famous video meme about an elephant painting.	477
N_EmmaWatsonMagazine	Emma Watson posed for Crash Magazine.	98
N_Englandback	Bank of England expected to cut interest rates to 1.5% or less. The lowest in the 315-year history.	152
N_Georgetiller	Late term abortion Dr. George Tiller shot to death at his church.	14495
N_Giantcoconutcrab	Information and pictures of a giant coconut crab.	59
N_HamsterOnAPiano	Reviews and reader's emotion for a video meme 'Hamster on a piano'.	1154
N_Havard	prominent black havard prof arrested for breaking into his own house (he was locked out.).	225
N_heathLedger	Heath ledger is died.	3503
N_IranProtestVideo	News and status update about Iran protest vidoe.	22653
N_Ivancamerondied	David Cameron's special son Ivan died.	131
N_JayceeDugard	Abductee had two children with captor, authorities say: Jaycee Dugard, now 29, was kidnapped.	1838
N_JenniferHudson	Shocking news about Jennifer Hudson's mom and brother being shot and killed.	369
N_Jhonbrain	A celtic star john hartson has a brain cancer.	302
N_josefFritzl	Update and emotions for Josef Fritzl's trial case. He's guilty for rape and incest.	4041
N_LockBumping	Reviews and viewers' emotion for a video meme, 'Lock bumping'.	13
N_NikonD300s	Information and reviews about a camera, Nikon D300s.	2511
N_ObamaFishing	Obama's Montana To-Do List: Discuss Health Care, Go Fly Fishing.	63
N_ObamaFly	Obama Swats a Fly Like a Boss During an Interview and related video.	14992
N_OJSimpson	A jury of 18 has been sworn in to hear the O.J. Simpson robbery case.	633
N_palmPre	Information and reviews about a pocket PC, palm pre.	7345
N_Peanutrecall	Rio Pluma LLC is Reissuing Recall of Peanut Products. They may be Contaminated with Salmonella.	151
N_PlaxicoBurress	Tweets about one episodes of Plaxico Burress for gun shooting.	1329
N_pregnantMan	Thomas Beatie, the transgender man who was born a woman, just welcomed his second child to the world.	3473
N_PrinceChunkCat	Prince chunk, the world's biggest cat.	53
N_PspGo	Information and reviews about a mobile device, psp go.	13458
N_SarahJessicaSurrogate	Sarah Jessica Parker expecting twins via surrogate.	1891
N_Sicko	Reviews and status updates for comedy drama film 'Sicko'.	469
N_SquareWatermelon	Tweets about an existing square shaped watermelon.	1872
N_surrogateMom	Melbourne Gay couple have twins by Indian surrogate.	36
N_Toughbook30	Information and reviews about a notebook, Toughbook 30.	131
N_Turkishcrash	Nine dead, 50 injured in Turkish Airlines passenger jet crash at Amsterdam.	875
N_twittersummize	Twitter bought Summize.	2849
N_Vanessa	Mother-of-two nursery worker arrested in child porn probe: Vanessa George.	159
N_Vince	News about a serious murderer.	10
N_WesternSpaghetti	Twitters about a video meme, Video: Western Spaghetti by team PES.	222
N_westNile	West Nile Virus Found in Knox County Mosquito Sample.	4961
