# Supplementary material for: Rumor Detection over Varying Time Windows
Source: PLoS One. 2017 Jan 12;12(1):e0168344. doi: 10.1371/journal.pone.0168344 (PMC5230768; doi:10.1371/journal.pone.0168344)
Supplement: S2 Table — (PDF) [file pone.0168344.s003.pdf]

S2 Table. Description of rumor events used for analysis.

Name	Description	#Tweets
R_alligator	Alligators live in sewer.	282
R_AsparagusCancer	Asparagus is a cancer cure.	88
R_barneyCocain	Barney Frank Snorts Cocaine.	25
R_Bigfoot	2 persons find the dead body of Bigfoot in Georgia.	505
R_breastInfestation	Tweets about a video meme that shows larvae in the breast.	113
R_Carmen	A rumor about chain mail to avoid curse of Carmen Winstead.	48
R_CellphoneGasExplosion	Use of cell phone at gas stations causes explosions.	20
R_Chupacabra	A legendary animal, Chupacabra, is found.	517
R_CookCellphone	Viral video and information that you can cook popcorn with mobile phones.	24
R_DennisKucinichUFO	Dennis Kucinich saw UFO.	42
R_DeodorantCancer	Deodorant can cause cancer.	258
R_DietCokeBacon	There's a diet coke with bacon flavour.	81
R_Dork	A word 'dork' means whale's penis.	916
R_Duckquack	A duck's quack doesn't echo, and no one know why.	718
R_Earwig	Be careful of earwigs, they can get in your ear and burrow through into your brain.	102
R_emmaWatson	Emma Watson died in car accident.	103
R_EricssonFreeLaptop	Rumors about Sony Ericsson free laptop.	125
R_GiantCatfish	There is really big catfish eating human.	304
R_GoogleSkype	Google will buy Skype.	507
R_HallmarkPostcarVirus	Information about the Postcard computer virus.	171
R_harrisonFord	Harrison Ford is died.	414
R_Hercules	Hercules, the world's biggest dog.	22
R_HIVKetchup	A man was caught putting his blood that HIV positive in bottles.	628
R_hydrogen	Hydrogen peroxide as a cancer treatment.	59
R_IphoneNano	Iphone Nano will be launched.	2715
R_IphoneOLED	Iphone with OLED display will be launched.	1439
R_Ipod64	Ipod with 64gb capacity will be soon launched.	315
R_Jamie	Jamie Lee Curtis is a hermaphrodite.	77
R_jeffGoldblum	Jeff Golblum died.	47
R_KayneWestKingOfPOP	Kayne West said that 'no one can match his sales' and he is the 'new King of Pop'.	116
R_KoreanFanDeath	Tweets mentioning Korean fan death rumors.	1006
R_LadyGaga	Lady Gaga is a hermaphrodite.	4820
R_ListerinMsquito	Listerine can deter mosquitoes.	240
R_ManEatingGoonch	Killer Catfish Goonch eats Human Flesh.	112
R_McCainManchurian	John McCain is the Manchurian Candidate.	95
R_MeganFox	Megan Fox was originally a guy that had a sex change.	51
R_MentosCokeDeath	A viral video and information that combination of coke and mentos can be possibly fatal.	61
R_MidgetPC	Information about IT product, midget PC.	17
R_MileyCyrus	Miley Cyrus is died.	142
R_Montauk	Urban legend of a monster.	2771
R_Mountainsperm	Mountain dew is not good for sperm.	123
R_NotCallRegi	Remember cell phone's go public next month 888-382-1222 to avoid cell phone telemarketers.	846
R_obamaanti	Obama is muslim and anti christ.	3971
R_obamaNationalDay	Obama cancelling National Day of Prayer.	56
R_obamaNaturalBorn	Obama is not a natural born citizen.	2157
R_ObamaOnePercent	"The top 1% income earners had a pretty good run of it. They will pay higher taxes under Obama."	121
R_onionIpod	Onion can charge Ipod.	579
R_PepsiAIDS	Pepsi can filled with AIDS blood.	41
R_ReversePIN	Entering your PIN number backwards at an ATM summons the police.	129
R_SarahPalin	Sarah Palin thinks dinosaurs existed 4000 years ago.	233
R_SarahPalinSambo	Sarah Palin called Obama and Hillary Sambo and the bitch.	14
R_SodaCanRatUrine	Rat urine in soda can.	19
R_SpermBank	Sperm bank where they have hot nurses who pay you to give you a hand job.	14
R_steorn	Information about Steorn's free energy machine.	129
R_SushiBrainWorm	Picture of maggots in brain because of eating raw fish.	15
R_SwinePork	Swine flu can be propagated by pork.	25974
R_sineZombie	Zombie is becoming fact with the swine flue outbreak.	4807
R_ToothFairy	The tooth fairy teaches children that they can sell body parts for money.	342
R_Xbox720	Xbox720 will be launched.	1012
R_ZunePhone	Zunephone will be launched.	604
