# Supplementary material for: Rumor Detection over Varying Time Windows
Source: PLoS One. 2017 Jan 12;12(1):e0168344. doi: 10.1371/journal.pone.0168344 (PMC5230768; doi:10.1371/journal.pone.0168344)
Supplement: S3 Table — Kurtosis is a measure of how much a given data is heavy or light-tailed compared to a normal distribution. Data with high kurtosis likely to have heavy tails or outliers. Skewness is a measure of symmetry. In definition column, a term ‘SD’ mean for standard deviation. (PDF) [file pone.0168344.s004.pdf]

**S3 Table. User features**

| Symbol | Definition | Symbol | Definition | Symbol | Definition |
|---|---|---|---|---|---|
| $\kappa_{fo}$ | Kurtosis of #followers | $\kappa_{fr}$ | Kurtosis of #friends | $\kappa_t$ | Kurtosis of #tweets |
| $S_{fo}$ | Skewness of #followers | $S_{fr}$ | Skewness of #friends | $S_t$ | Skewness of #tweets |
| $m_{fo}$ | Minimum of #followers | $m_{fr}$ | Minimum of #friends | $m_t$ | Minimum of #tweets |
| $q25_{fo}$ | 25% quantile of #followers | $q25_{fr}$ | 25% quantile of #friends | $q25_t$ | 25% quantile of #tweets |
| $med_{fo}$ | Median of #followers | $med_{fr}$ | Median of #friends | $med_t$ | Median of #tweets |
| $q75_{fo}$ | 75% quantile of #followers | $q75_{fr}$ | 75% quantile of #friends | $q75_t$ | 75% quantile of #tweets |
| $M_{fo}$ | Maximum of #followers | $M_{fr}$ | Maximum of #friends | $M_t$ | Maximum of #tweets |
| $\mu_{fo}$ | Average of #followers | $\mu_{fr}$ | Average of #friends | $\mu_t$ | Average of #tweets |
| $\sigma_{fo}$ | SD of #followers | $\sigma_{fr}$ | SD of #friends | $\sigma_t$ | SD of #tweets |

Kurtosis is a measure of how much a given data is heavy or light-tailed compared to a normal distribution. Data with high kurtosis likely to have heavy tails or outliers. Skewness is a measure of symmetry. In definition column, a term ‘SD’ mean for standard deviation
