# Supplementary material for: Rumor Detection over Varying Time Windows
Source: PLoS One. 2017 Jan 12;12(1):e0168344. doi: 10.1371/journal.pone.0168344 (PMC5230768; doi:10.1371/journal.pone.0168344)
Supplement: S4 Table — In definition column, E, F, and D mean for extended network, friendship network and diffusion network, respectively. (PDF) [file pone.0168344.s005.pdf]

S4 Table. Network features

Symbol	Definition	Symbol	Definition
V_e	# Nodes in E	V_f	# Nodes in F
E_e	# Edges in E	E_f	# Edges in F
NI_e	# Nodes without incoming edges in E	NI_f	# Nodes without incoming edges in F
NO_e	# Nodes without outgoing edges in E	NO_f	# Nodes without outgoing edges in F
I_e	# isolated nodes in E	I_f	# isolated nodes in F
pNI_e	Percent of nodes without incoming edges in E	pNI_f	Percent of Nodes without incoming edges in F
pNO_e	Percent of nodes without outgoing edges in E	pNO_f	Percent nodes without outgoing edges in F
pI_e	Percent of isolated nodes in E	pI_f	Percent of isolated nodes in F
V_{el}	# Nodes in LCC of E	V_{fl}	# Nodes in LCC of F
E_{el}	# Edges in LCC of E	E_{fl}	# Edges in LCC of F
AD_{el}	Average degree of nodes in LCC of E	AD_{fl}	Average degree of nodes in LCC of F
AC_{el}	Average clustering coefficients of LCC of E	AC_{fl}	Average clustering coefficients of LCC of F
D_{el}	Density of LCC of E	D_{fl}	Density of LCC F
V_d	# Nodes in D	LTH	# Edges, whose tail node has lower #followers in D
E_d	# Edges in D		(# Information diffusion from lower degree user)
NI_d	# Nodes without incoming edges in D	HTL	# Edges, whose tail node has higher # followers in D
NO	# Nodes without outgoing edges in D		(# Information diffusion from lower degree user)
I_d	# isolated nodes in D	pLTH	Fraction of LTH among information diffusion
pNI_d	Percent of nodes without incoming edges in D	pHTL	Fraction of HTL among information diffusion
pNO_d	Percent of nodes without outgoing edges in D		
pI_d	percent of isolated nodes in D		
V_{dl}	# Nodes in LCC of D		
E_{dl}	# Edges in LCC of D		
AD_{dl}	Average degree of nodes in LCC of D		
AC_{dl}	Average clustering coefficients of LCC of D		
D_{dl}	Density of LCC of D		

In definition column, *E*, *F*, and *D* mean for extended network, friendship network and diffusion network, respectively.
