# Supplementary material for: Rumor Detection over Varying Time Windows
Source: PLoS One. 2017 Jan 12;12(1):e0168344. doi: 10.1371/journal.pone.0168344 (PMC5230768; doi:10.1371/journal.pone.0168344)
Supplement: S5 Table — Symbols are for parameters of suggested PES model. (PDF) [file pone.0168344.s006.pdf]

S5 Table. Temporal features

Symbols	Definition
N	Total population of available users
β	Probability of infection
n_b	Starting time of breaking news
S_c	Strength of external shock at birth (time n_b)
ϵ	Background noise
p_a	Strength of interaction periodicity
p_s	Interaction periodicity offset
q_a	Strength of external shock
q_p	Periodicity of external shock
q_s	External shock periodicity offset

Symbols are for parameters of suggested PES model.
